# Peptide Epitope Hot Spots of CD4 T Cell Recognition Within Influenza Hemagglutinin During the Primary Response to Infection

**DOI:** 10.3390/pathogens8040220

**Published:** 2019-11-05

**Authors:** Zackery A. G. Knowlden, Katherine A. Richards, Savannah A. Moritzky, Andrea J. Sant

**Affiliations:** David H. Smith Center for Vaccine Biology and Immunology, Department of Microbiology and Immunology, University of Rochester Medical Center, Rochester, NY 14642, USA; Zackery_Knowlden@URMC.Rochester.edu (Z.A.G.K.); Katherine_skelly@urmc.rochester.edu (K.A.R.); Savannah_Moritzky@URMC.Rochester.edu (S.A.M.)

**Keywords:** influenza, CD4 T cell, epitopes, hemagglutinin

## Abstract

Antibodies specific for the hemagglutinin (HA) protein of influenza virus are critical for protective immunity to infection. Our studies show that CD4 T cells specific for epitopes derived from HA are the most effective in providing help for the HA-specific B cell responses to infection and vaccination. In this study, we asked whether HA epitopes recognized by CD4 T cells in the primary response to infection are equally distributed across the HA protein or if certain segments are enriched in CD4 T cell epitopes. Mice that collectively expressed eight alternative MHC (Major Histocompatibility Complex) class II molecules, that would each have different peptide binding specificities, were infected with an H1N1 influenza virus. CD4 T cell peptide epitope specificities were identified by cytokine EliSpots. These studies revealed that the HA-specific CD4 T cell epitopes cluster in two distinct regions of HA and that some segments of HA are completely devoid of CD4 T cell epitopes. When located on the HA structure, it appears that the regions that most poorly recruit CD4 T cells are sequestered within the interior of the HA trimer, perhaps inaccessible to the proteolytic machinery inside the endosomal compartments of antigen presenting cells.

## 1. Introduction

Influenza hemagglutinin (HA) is a major target of influenza vaccination because of the importance that this molecule plays in mediating binding and infection of influenza virus on host cells. Because of this function, antibodies elicited by most influenza vaccine strategies have the potential to provide sterilizing immunity to influenza infection in the host (reviewed in [1,2,3,4,5,6,7]). CD4 T cells specific for HA are also critical in the protective antibody responses to infection and vaccination because of their role in the germinal center response, high affinity antibody production and B cell memory (reviewed in [8,9,10]). Our studies show that in human vaccine responses to influenza, the serum antibody responses to HA are correlated with the elicitation of CD4 T cells specific for HA peptide epitopes and not those from other viral proteins contained in the vaccine, such as NP (nucleoprotein) [11]. Similarly, in animal models of influenza infection, recall of memory CD4 T cells specific for HA, but not NP, is associated with neutralizing antibody response to HA [12]. The preceding studies suggest that, although the human CD4 T cell repertoire is highly diverse with respect to reactivity to influenza viral proteins [13,14,15,16,17,18,19,20,21], understanding the specificity of CD4 T cells towards the HA protein is particularly important.

There have been a number of studies that have reported individual HA epitopes that are recognized by CD4 T cells in both human and mouse models of infection and vaccination (reviewed in [22,23]). However, published studies have often been limited to the analyses of particular regions of HA, chosen based on common sequences in diverse subtypes of influenza [24], or shared epitopes between seasonal and potentially pandemic strains of influenza [16,20,25,26,27,28,29]. A subset of peptides in HA have also been studied because they are presented by particular allelic forms of human HLA (Human Leukocyte Antigen) class II molecules that are well-suited for class II-peptide tetramer analyses [14,28,30,31,32]. Because of our interest in the full potential repertoire of influenza-specific CD4 T cells, our laboratory has used unbiased epitope scanning methods to identify the entire array of peptides recognized by CD4 T cells in the primary response in mice to influenza infection or vaccination. These studies have involved a diverse set of mouse strains, chosen in part to explore the role of MHC (Major Histocompatibility Complex) class II polymorphism in selection of the immunodominance hierarchy [33,34], the relationship between epitope selection in CD4 T cell responses to infection vs. vaccination [35], CD4 T cell epitope selection during homing to the lung after infection [36,37], partitioning of CD4 T cells into follicular helper cells versus effector cell lineages after infection and vaccination [38], and epitope mapping for identification of human CD4 T cell epitopes via HLA-DR transgenic mice [39,40]. Collectively, these studies have revealed a tremendous breadth in the CD4 T cell response to influenza that includes specificities derived from HA, NA, M1, NP, and the polymerase proteins.

In the study reported here, we analyzed the peptide epitope distribution of HA-specific CD4 T cells elicited by influenza infection from a panel of independent strains of mice. These mice were chosen because, based on unique MHC class II molecules that they express, they will each select an independent CD4 T cell peptide repertoire from the HA protein. Our studies revealed that the epitopes selected for CD4 T cells in the primary response are localized to a limited number of “hot spots” within the HA protein and that there are corresponding “dead zones” that recruit very few CD4 T cells. The basis of the immunodominance in the influenza HA protein is not fully understood at this time but is important to understand in developing vaccine strategies for influenza virus. 

## 2. Results

### 2.1. HA Epitope Dominance in the Primary Response to Influenza Infection

To map individual epitopes from the influenza HA protein, common inbred mice expressing different murine MHC class II molecules (thus having the potential to present different HA-derived peptides) or transgenic mice, expressing human HLA-DR or HLA-DQ molecules, were infected with A/New Caledonia/20/99 H1N1 virus, a recent human isolate which replicates in mice [41] but that is not highly pathogenic in any of the strains tested. At day 10–13 post-infection, peptide epitopes recognized by the elicited CD4 T cells were identified by a sequential method involving a peptide pooling matrix, which we described in detail previously [39,40]. In this method, single overlapping peptides representing the entire translated HA protein are pooled in sets of 9–10 different peptides and arrayed in a matrix strategy originally described by Tobery and colleagues for identification of T cell specificities [42]. Spleen cells from infected mice were used as a source of primed CD4 T cells, enriched by negative paramagnetic bead isolation, as described in Materials and Methods. CD4 T cells were tested for reactivity to the HA peptide pools that were arrayed in a matrix design and quantified using cytokine EliSpot assays. IL-2 producing CD4 T cells were used to quantify reactivity to influenza epitopes from spleen because in some strains of mice, this is the dominant cytokine expressed particularly in the spleen ([41] and data not shown). In addition, in contrast to cells localized to the lung, that are enriched for IFN-γ [36,37], IL-2 is reliably expressed by CD4 T cells elicited by infection that localize to secondary lymphoid tissue [34]. Use of peptides and splenic CD4 T cells allowed all potential HA epitopes recognized by the host CD4 T cells to be presented by APC and available to recall the primed CD4 T cells. The primed CD4 T cells and syngeneic splenocytes from naïve mice or MHC class II expressing fibroblasts (for HLA-DR1 transgenic mice), used as a source of APC, were co-incubated with the peptide pools for 16–20 h. Pools tested as negative were eliminated from further analyses. Candidate peptides in the positive rows and columns were then tested as single peptides. Through this sequential, iterative process, the single CD4 T cell peptide epitopes derived from HA were identified.

Supplemental Table S1 lists each of the peptides in the overlapping peptide library that was used in the initial epitope screening for each mouse strain. Supplemental Table S2 shows the peptide specificities of all the mouse strains that were analyzed with the indicated average cytokine spot count from at least three replicate experiments. We classified peptides that elicited at least 125 cytokine-producing cells as positive for this study because these were the most reliably quantified in replicate, independent assays. These curated data are shown in Table 1, which also denotes the mouse strain and MHC class II haplotype and allelic forms of class II expressed. Because of the large range in responses to different peptide epitopes, we subdivided the positive epitopes into two groups: subdominant (125–300 cytokine-producing cells) and dominant (>300 cytokine-producing cells). The typical range in HA-reactive epitope-specific CD4 T cells in any given mouse strain ranged from 125–400 spots, although an occasional epitope recruited more than 600 cytokine-producing CD4 T cells. 

**Figure 1:**
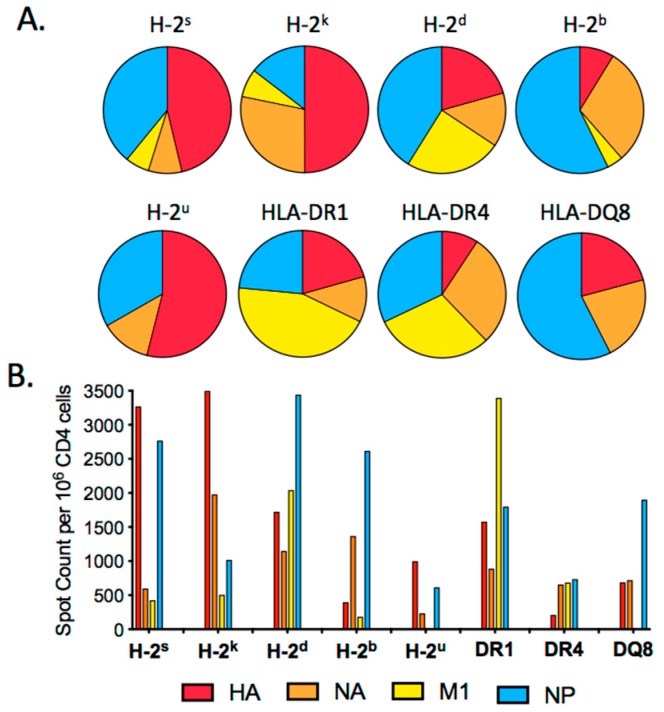
Pattern of CD4 T cell reactivity to influenza viral proteins following primary infection. Influenza reactivity to influenza viral proteins following primary infection with A/New Caledonia/20/99 was measured by IL-2 EliSpot assays as spots per million CD4 T cells to HA (red), NA (orange), M1 (yellow), and NP (blue), where the total number of CD4 T cells producing IL-2 specific for defined epitopes within each viral protein were summed. In panel **A** the immunodominance hierarchies for each mouse strain are illustrated as pie charts, with the fraction of the response dedicated to each viral protein is indicated. Responses for the M1 protein were not assessed in the H-2u and HLA-DQ8 mouse models and therefore their immunodominace hierarchies as illustrated are limited to HA, NA and NP. In panel **B** the total spot counts are indicated as bar graphs for each MHC haplotype in the strains of mice analyzed.

It is interesting to note that the number of HA-derived epitopes recognized by CD4 T cells from different strains of mice varied considerably (see Supplemental Table S2). For example, H-2^b^ mice, expressing I-A^b^, recruited very few CD4 T cells specific for HA, all below 125 spots per million, while other mice, including A/J mice, expressing I-A^k^ and I-E^k^ class II molecules, elicited CD4 T cells specific for greater than fifteen epitopes in HA, six of which were above the subdominant threshold. Although mice tested in the current studies differ both in background genes and in their MHC class II haplotype, our published studies involving B10.S (H-2^s^) and B10 (H-2^b^) mice that are identical in non-MHC background genes recruit strikingly different epitope specificities [33,34]. These studies, as well as our unpublished data with many inbred strains of mice, suggest that the major factor determining CD4 T cell epitope specificity and abundance after influenza infection is the MHC class II molecules expressed in the host. All strains of mice used in the current study elicited robust CD4 T cell responses to other viral proteins after influenza infection, including those specific for M1, NA, and NP ([22,34,36,37,38,39,40,43], as shown in Figure 1). 

### 2.2. Distribution of Dominant and Subdominant CD4 T Cell Epitopes in the HA Protein Sequence

**Figure 2:**
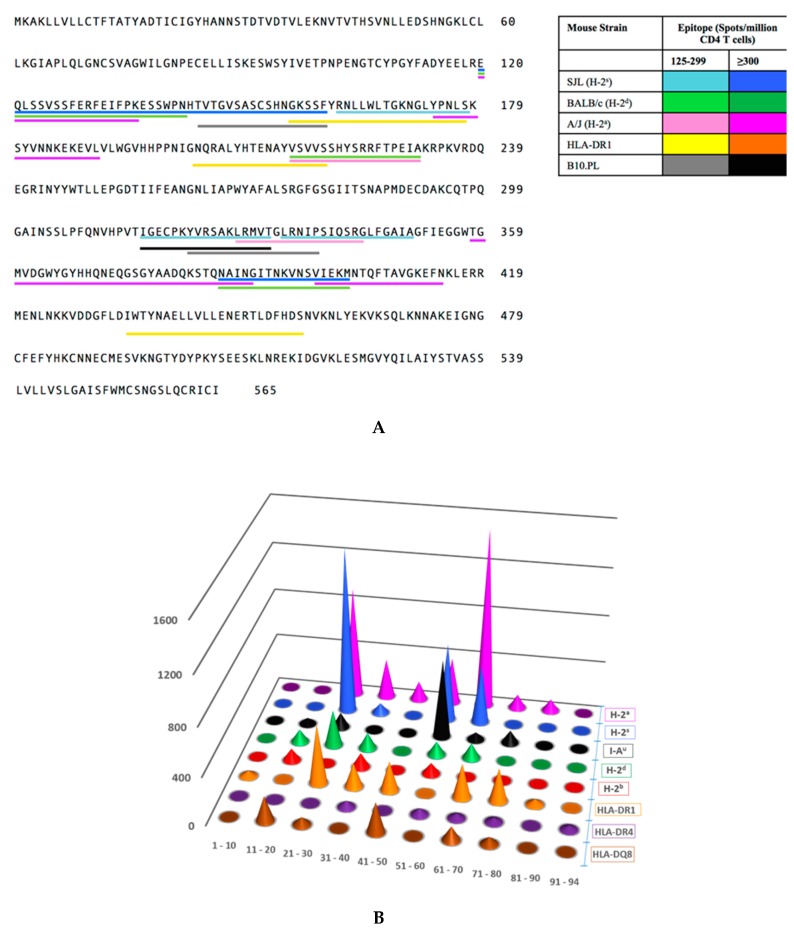
CD4 T cell epitopes within the hemmaglutinin protein. Panel (**A**) shows the amino acid sequence from N- to C- terminus including the leader sequence for H1N1 A/New Caledonia/20/99. The underlined segments indicate the amino acids that comprise CD4 T cell peptide epitopes within HA protein that elicit >125 spots/million when measured by cytokine EliSpots. Each color corresponds to a different mouse strain (each with a different MHC haplotype) as indicated, with the darker hue representing ^3^300 spots/million and the lighter hue representing 125–299 spots/million. Shown to the lower right is the key to the strain designation and color indicators. Responses from HLA-DR4 and C57BL/10 mice are not included in this illustration because their epitopes were below the positive threshold. In Panel (**B**), the reactivity to individual CD4 T cell epitopes, within HA, as measured by IL-2 EliSpot assays as spots per million CD4 T cells (See Table 1), were summed for each strain of mouse examined and placed into the “decades” of peptides spanning the entire sequence of the HA protein as described in results. All decades contain ten peptides except for the last set (peptides 91–94). Cumulative reactivity for each decade is represented as a cone with height corresponding to the summed spots per million (Y-axis) for each strain of mouse. Cones are colored for each MHC haplotype or MHC class II molecule for the various strains of mice utilized for this study as shown on the Z-axis.

After compiling all of the CD4 T cell immunodominance hierarchy data, we examined the distribution of epitopes within the HA sequence that met the threshold of a dominant or subdominant epitopes, shown in Supplemental Table S2. These data are shown in Figure 2A. Interestingly, this data representation shows that the peptide epitopes that elicited CD4 T cells were not distributed equally across the HA protein. Instead, they tended to cluster in several distinct regions, which we have termed “hot spots” of reactivity. The biggest clusters of CD4 T cell epitopes in HA, recognized by multiple strains of mice were clustered between amino acids 120–190 and 316–414. Other segments of HA, including amino acid 1–119, 232–315, and 457–565 were relatively devoid of CD4 T cell epitopes, with just an occasional minor peptide epitope represented in these segments. We have termed these regions “dead zones” of reactivity.

**Figure 3:**
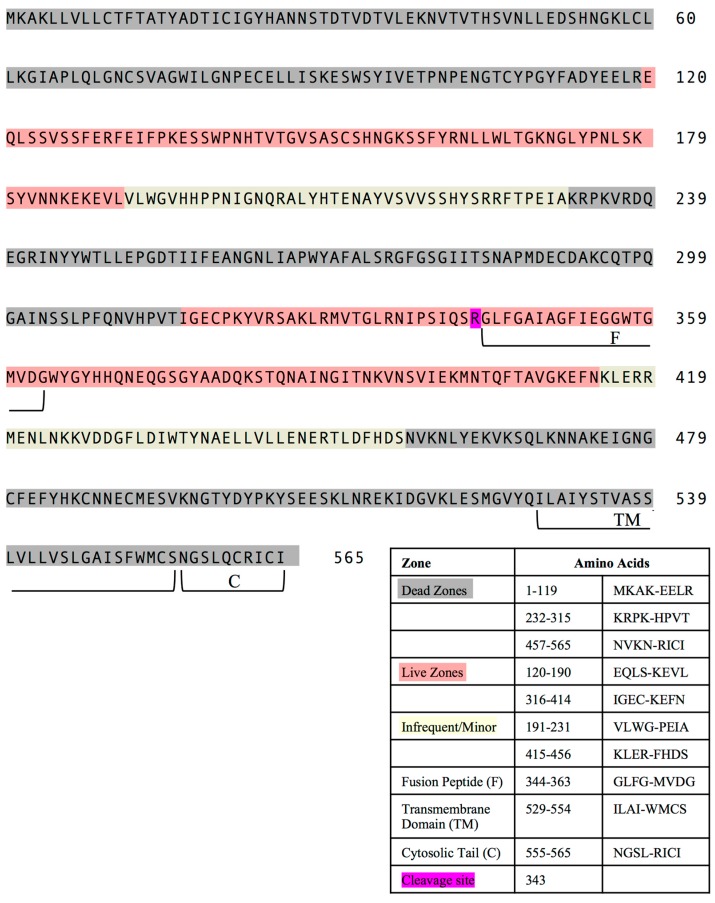
**Stretches of the HA sequence that have distinct regions of CD4 T cell epitope dominance.** The HA sequence is shown highlighted in regions that contain many dominant epitopes (shown in red), minor epitopes (shown in cream) or devoid of epitopes (shown highlighted in dark grey).

We then analyzed these data in a graphical display (Figure 2B). There are approximately 100 16–17 mer peptides in the overlapping array provided by the supplier, numbered from amino acid 1–94, from amino (‘N”)- to carboxy (C)-terminus (Supplemental Table S2). The peptides were grouped by each decade of HA amino acid sequence (e.g., peptides 1–10, peptides 11–20) (from N to C terminus), indicated on the X-axis. The total CD4 T cell responses, quantified as cytokine spots per million, reflecting the abundance of HA-reactive CD4 T cells in each strain, are depicted by the height of the “cones” on the Y-axis, with each cone depicted by color based on the strain of mouse and the MHC molecules expressed, shown to the left Z-axis. This display allows ready visualization of the distribution and magnitude of the CD4 T cell epitopes, and their clustering across the HA protein. From depicting the immunodominance data in this way, it is clear that among the eight strains of mice studied, there are dominant regions of CD4 T cell recognition within HA. Peptides contained within decade 21–30 and the two decades 51–70, shown in Figure 2, appear to contain the vast majority of the CD4 T cell epitopes. Conversely, there are extended regions of HA that are relatively devoid of CD4 T cell epitopes, notably the first two decades (1–20) and final two decades. The segments of high and very low reactivity, as well as the segments that have only minor epitopes are indicated on the amino acid sequence of the HA protein in red, dark grey, and cream, respectively, in Figure 3. 

### 2.3. Location of Live and Dead Zones on the HA Structure

**Figure 4:**
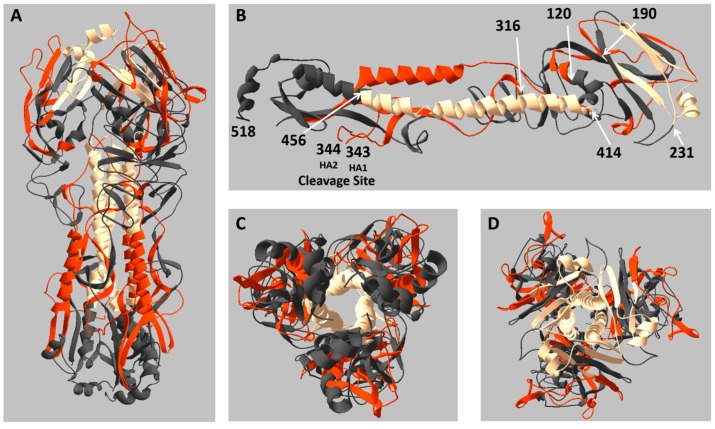
Localization of regions of HA that are enriched for or that lack CD4 T cell epitopes. Regions of CD4 T cell reactivity were mapped onto the trimeric structure of HA, with “Live” depicted in red, “Minor” in cream, and “Dead” in dark grey, categorized based on the data shown in Figure 2 are shown in (**A**). Amino acid residues at transition points between areas of differing reactivity are indicated on the HA monomer (**B**), as well as the HA cleavage site (amino acids 343, 344) and the carboxy terminus of the resolved structure (amino acid 518). The membrane proximal region (**C**, bottom view) of the trimer, as well as the head region (**D**, top view), are provided as an alternate vantage point of the HA protein structure. The structure is adapted from the H1N1 A/Brevig Mission/1/1918 hemagglutinin HA0 crystal structure (PDB: 1RD8), a close analog of A/New Caledonia/20/1999.

From our analysis of the “zonal” positioning of CD4 T cell epitopes, we were able to determine regions of the HA protein that had tendencies that favored or disfavored the generation of peptide epitopes in the context of many different MHC molecules. Accordingly, we sought to understand where the zones of reactivity mapped onto the structure of the hemagglutinin protein (Figure 4A,C,D). For this analysis, we designated regions of reactivity in three categories: “live” (regions of high reactivity, colored in red); “dead” (regions of limited/no reactivity, colored in dark grey); and “minor” (regions of occasional CD4 T cell reactivity, colored in cream). As shown in the HA monomer in Figure 4B, live regions stretched from amino acid 120 to 190, and from 316 to 414. Dead regions run from the amino-terminus to amino acid 119, then from 232 to 315 and from 457 to the carboxy-terminus (position 518). The transmembrane domain and membrane proximal amino acids are not depicted in the structure, but are included in the dead zones. Regions of minor reactivity run from residues 191 to 231, as well as 415 to 456. The site where HA0 is cleaved to form HA1 and HA2 (amino acids 343 and 344) is also indicated. 

What becomes immediately clear from this rendition is the distinct structural segregation of the different zones of activity. The live regions, responsible for eliciting the majority of CD4 T cell epitopes, are located in regions that are highly structured, but also solvent exposed, suggesting that the stability of the tertiary structure coupled with accessibility to proteasomal processes is critical for the generation of CD4 T cell epitopes to HA. This feature is best illustrated by the alpha-helical domain in the stem region and the sheet structure in the head of the trimer. In contrast, the dead regions of HA are clustered in the membrane proximal region (Figure 4C), as well as in loosely structured regions between the head and stem (Figure 4A,B). The dead portion of HA found in the head of the trimer is in a well-ordered sheet structure, but is somewhat occluded by the overlaying red-colored sheet on the outside of the protein (Figure 4D), possibly inaccessible to proteases. The regions of HA designated as having minor reactivity are all relegated to the interior of the trimer, centralized in the triple-helical structure of the stem and the underlying base of the layered sheet structure of the head. It is likely that the regions of minor reactivity are under-represented as CD4 T cell epitopes due to both the highly ordered tertiary structure and the internal positioning within the HA protein.

## 3. Discussion

The studies reported here suggest that in the primary response to influenza infection, only subsets of the segments of HA are selected by the elicited CD4 T cell repertoire. It is reasonable to speculate that such selection is multifactorial. Selection of CD4 T cell responses in the host has been shown to reflect at least in part the sequential processes in antigen presentation, including antigen uptake into endosomal compartments of antigen presenting cells (APC), pH-induced unfolding, reduction of disulfide bonds, proteolytic release of antigenic peptide, acquisition of the peptide by host MHC class II molecules and editing by HLA-DM and, finally the export of the peptide:class II complex to the cell surface of the APC (reviewed in [44,45,46,47,48,49,50,51,52,53,54,55,56,57]). Factors that have been implicated in previous studies for selection of dominant epitopes by CD4 T cells include proteolytic processing, three dimensional structure, sensitivity to proteolytic enzymes, or biochemical features of peptide:MHC class II complexes [56,58,59,60,61,62,63]. Selection of CD4 T cells is also dependent on the T cell receptor repertoire in the host that can recognize the presented peptide:class II complexes by APC [64,65,66]. 

It is interesting to consider the mechanisms that may underlie the characteristic dominant “hot spots” and “dead zones” of HA, representing regions that are enriched for CD4 T cell epitopes or that have a paucity of them, respectively. First, it is possible that the segments of HA within the hot spots of reactivity have atypically broad ability to bind to MHC class II molecules. However, because of the diversity of hosts that recognize these segments, each possessing distinct MHC class II molecules with their own binding preferences (reviewed in [67,68]), this seems less likely to be a dominant determining factor in selection of epitopes from these regions. As discussed, it is possible that the multimeric nature of the HA trimer limits proteolytic access of some internal sequestered regions of the trimeric HA, as is shown for the regions of the HA trimer that contain very few epitopes indicated in Figure 4. This feature of multimeric proteins may be particularly important in MHC class II epitope selection. The hot spots, shown in Figure 4, suggest that they tend to be on the solvent exposed regions that may be particularly accessible to proteolytic enzymes. Interestingly, the element of access to proteolytic sensitivity in viral proteins has been implicated in selection of CD4 T cell immunodominant regions of HIV gp140 protein [69] and Zika virus C and E proteins [70], as well as in studies designed to generally predict antigenic site for CD4 T cell recognition [45]. 

Another element that may be involved in epitope selection of HA is sites of N-linked glycosylation. Although it is known that some glycopeptides can elicit CD4 T cells (reviewed in [71,72]), it is possible that the sites of complex or very large N-linked glycosylation may shield segments of HA from proteolysis, as has been observed for the endosomal proteins LAMP-1 and LAMP-2 [73] and synthetic peptides [74]. Alternatively, the N-linked carbohydrate may obstruct CD4 T cell receptor recognition. Either of these events would diminish the frequency of CD4 T cells that can be elicited, even if these peptides can bind to MHC class II molecules [75]. We did note that the high epitopic regions of HA were devoid of potential N-linked glycosylation sites, except at the boundaries of the antigenic peptides (data not shown). Clearly, more experiments are needed to dissect the basis for the selection of restricted regions of HA in the primary response to influenza. However, it is interesting to consider the possibility that accumulation of sites that allow N-linked carbohydrates to be added, a well-documented phenomena in drifted Influenza A viruses, speculated to occur to shield HA from recognition of protective antibody recognition [76,77,78] may also serve to diminish CD4 T cell epitope generation.

An additional issue that is relevant to our studies relates to the nature of the priming antigen presenting cell after infection, the cellular mechanisms involved in class II:peptide epitope generation and the natural substrate for processing of HA and presentation of HA-derived peptides. Several recent publications [79,80,81] suggested that influenza infection might select for a distinct set of CD4 epitopes than those elicited by vaccination through a pathway initiated by infected antigen presenting cells. These data suggested to us that protein vaccination strategies, used extensively to provide protective immunity to influenza might misdirect the CD4 T cells response to a distinct subset of epitopes from those elicited by infection, diminishing the ability of influenza vaccines to CD4 T cells be recruited into the response to infection. To address this important issue, we recently we formally compared CD4 T cell epitope selection in mice via influenza infection vs. vaccination with recombinant NP and HA [35] revealed that the multitude of individual NP and HA epitopes elicited by these two modes of priming were the same. Moreover, we found that protein vaccination leads to memory CD4 T cells that can be recalled by infection. Thus, in the influenza model in mice that we have used in this study, as well as an earlier study from our group [12], suggest that the primary source of HA used for antigen presentation after infection likely involves intact HA released by infected or dying cells, rather than infectious virions.

In addition to considering the mechanisms that might underlie the CD4 T cell immunodominance of selected regions of HA, it is interesting to consider implications of these studies to human CD4 T cell immunity to influenza. Examining the CD4 T cell HA epitope specificity in the primary responses to influenza vaccination and infection is not possible in humans because of the extremely low frequency of individual-influenza CD4 T cells specific for single epitopes [14,16,20,82], coupled with the low blood volumes that it is possible to sample in infants and young children [83]. Our sampling of circulating adult human CD4 T cells specific for broad segments of HA suggest that regions of high genetic conservation are somewhat dominant in the circulating memory population [11,20,84]. It seems likely that epitope preferences within HA initiated during the first encounter of the human host might be shifted in specificity over time, due to the repeated and periodic confrontations of humans to alternative influenza strains generated by antigenic drift and shift. There are highly conserved regions of HA that will likely be repeatedly encountered in humans over a lifetime, particularly in the membrane proximal stem domain. HA-derived antigenic peptides derived from these regions may continually boost and expand CD4 T cells for these highly conserved regions, while specificities elicited in the primary response may decay in their relative frequency over time. It will be of interest to determine whether immunodominance hierarchies in HA shift toward these highly conserved regions as human subjects age or as shift and drift occurs, eliminating the potential of altered or eliminated CD4 T cell epitopes to boost the CD4 T cell repertoire upon influenza vaccination or infection. 

## 4. Materials and Methods

### 4.1. Mice

The HLA-DR1 (B10.M/J-TgN-DR1) and HLA-DR4 (C57BL/6Tac-Abb<tm>TgNDR4) transgenic mice were obtained from D. Zaller (Merck) through Taconic Laboratories. The HLA-DQ8 (DQB.0302) transgenic mice were obtained from C. David [85]. B10.PL (B10.PL-H2^u^H2-T18^a^/(73NS)SnJ) mice were obtained from Jackson Laboratory. The DR1, DR4, DQ8, and B10.PL mice were bred and maintained in the specific pathogen free facility at the University of Rochester. Commercially available female C57BL/10, BALB/c, A/JCr, and SJL/JCr were purchased from National Cancer Institute-Frederick (Frederick, MD). All mice were used at 2–6 months of age.

### 4.2. Ethics Statement 

All mice were maintained in the specific-pathogen free facility at the University of Rochester Medical Center according to the institutional guidelines. All animal protocols used in this study adhere to the AAALAC International, the Animal Welfare Act, and the PHS Guide and were approved by the University of Rochester Committee on Animal Resources, Animal Welfare Assurance Number A3291-01. The protocol under which these studies were conducted was originally approved 4 March 2006, (protocol no. 2006-030) and has been reviewed and re-approved every 36 months with the most recent review and approval 23 January 2018.

### 4.3. Peptides 

17-mer peptides overlapping by 11 amino acids to encompass the entire sequence of the HA of influenza virus A/New Caledonia/20/99 (NR-2602) were obtained from BEI Resources, ATCC. The peptides were reconstituted at 10 mM in PBS, with or without added dimethyl sulfoxide, to increase solubility of hydrophobic peptides, and 1 mM dithiothreitol, for cysteine containing peptides. Working stocks (1 mM) were prepared in complete DMEM, (Dulbecco’s Minimal Essential Media), sterilized and stored at −20 °C, as were concentrated stocks. 

### 4.4. Infections, Tissue Harvest and Preparation of CD4 T Cell Enriched Cell Populations

A/New Caledonia/20/99 virus was produced as we have previously described [34,39]. Mice were infected intranasally at 20,000–100,000 EID_50_ in 30 ul of phosphate buffered saline (PBS) after being anesthetized by intraperitoneal injection with tribromoethanol (Avertin; 250–300 ul per mouse, normalized to weight). Spleens were excised at 10–13 days post-infection. For cytokine EliSpots, spleen cells were pooled from infected mice and used as the source of CD4 T cells. Cells were depleted of red blood cells (RBC) using ACK Lysis Buffer (0.15 M NH_4_Cl, 1 mM KHCO_3_, and 0.1 mM Na_2_-EDTA in H_2_O, pH 7.2). After washing, cells were depleted of B cells, CD8 cells, and class II positive cells using MACS negative selection (Miltenyi Biotec), according to the manufacturer instructions.

### 4.5. EliSpot Assays 

EliSpot assays were performed as previously described [39]. Briefly, 96-well filter plates (Millipore) were coated with 2 ug/ml purified rat anti-mouse IL-2 (clone JES6-1A12, BD Biosciences) in PBS overnight at 4 °C, washed with media to remove unbound antibody and incubated with 100 ul media per well for 1 h to block non-specific binding. Isolated CD4 T cells were co-cultured with syngeneic spleen cells or transfected fibroblast antigen presenting cell (APC) (for HLA-DR1 transgenic mice [39]) and peptides for 16–20 h at 37 °C and 5% CO_2_. The cells were removed from the plates, and the plates were washed with wash buffer (1X PBS, 0.1% Tween-20). Biotinylated rat anti-mouse IL-2 (clone JES6-5H4, BD Biosciences) was added at a concentration of 2 ug/ml, 50 ul/well, in wash buffer with 10% FBS (Fetal Bovine Serum) and incubated at room temperature for 30 min. The plates were washed again and streptavidin-conjugated alkaline phosphatase (Jackson Immuno Research) was added at a dilution of 1:1000 in wash buffer with 10% FBS, 50 ul/well, and incubated for 30 min at room temperature. The plates were and developed using Vector Blue substrate kit III (Vector Laboratories, City, CA, USA) prepared in 100 mM Tris, pH 8.2. After drying, quantification of spots was performed with an Immunospot reader series 5.2, using Immunospot software, version 5.1.

### 4.6. Structural Analyses

The structure of hemagglutinin (1RD8) [86] was analyzed for the internal positioning of CD4 T cell epitopes. This protein structure was acquired from the Protein Data Base (PDB ID indicated) and modified by color in Swiss-Pdbviewer to indicate by color the regions of HA that correspond to the abundance of CD4 T cell epitopes.

## Figures and Tables

**Table 1:** Mouse strain dependent immunodominance hierarchy in CD4 T cell epitopes specific for HA from A/New Caledonia/20/99. Each mouse strain with HA epitopes >125 spots/million CD4 T cells is shown. Also indicated for each strain is the MHC haplotype and MHC class II molecules expressed. On the far right is the average cytokine spots/million for each epitope from at least 3 independent experiments. These epitopes were those selected for the data shown in Figure 1 and Figure 2. The peptides in bold represent those with 300 spots/million.

Mouse Strain	MHC Haplotype	Peptide	Amino Acids	Sequence	Spots Per 10^6^ Cells
A/J	H-2^a^	**HA p21**	**120–136**	**120 EQLSSVSSFERFEIFPK 136**	**474**
		**HA p30**	**174–190**	**174 YPNLSKSYVNNKEKEVL 190**	**374**
		HA p37	215–231	215 VSVVSSHYSRRFTPEIA 231	236
		HA p56	328–344	328 LRMVTGLRNIPSIQSRG 344	271
		**HA p61**	**358–374**	**358 TGMVDGWYGYHHQNEQG 374**	**507**
		**HA p64**	**375–391**	**375 SGYAADQKSTQNAINGI 391**	**351**
		**HA p68**	**398–414**	**398 VIEKMNTQFTAVGKEFN 414**	**640**
B10.PL	I-A^u^	HA p25	144–160	144 TVTGVSASCSHNGKSSF 160	124
		**HA p54**	**316–332**	**316 IGECPKYVRSAKLRMVT 332**	**369**
		HA p55	322–338	322 YVRSAKLRMVTGLRNIP 338	289
HLA-DQ8	DQ8	HA p14	78–94	78 ILGNPECELLISKESWS 94	136
		HA p48	280–296	280 GIITSNAPMDECDAKCQ 296	155
HLA-DR1	DR1	HA p27	156–172	156 GKSSFYRNLLWLTGKNG 172	167
		HA p28	162–178	162 RNLLWLTGKNGLYPNLS 178	163
		HA p35	203–219	203 NQRALYHTENAYVSVVS 219	159
		HA p74	434–450	434 IWTYNAELLVLLENERT 450	128
		HA p75	440–456	440 ELLVLLENERTLDFHDS 456	146
BALB/c	H-2^d^	HA p21	120–136	120 EQLSSVSSFERFEIFPK 136	146
		HA p22	126–142	126 SSFERFEIFPKESSWPN 142	151
		HA p37	215–231	215 VSVVSSHYSRRFTPEIA 231	130
		HA p66	386–402	386 NAINGITNKVNSVIEKM 402	124
SJL	H-2^s^	**HA p21**	**120–136**	**120 EQLSSVSSFERFEIFPK 136**	**306**
		**HA p22**	**126–142**	**126 SSFERFEIFPKESSWPN 142**	**663**
		**HA p23**	**132–148**	**132 EIFPKESSWPNHTVTGV 148**	**300**
		**HA p25**	**144–160**	**144 TVTGVSASCSHNGKSSF 160**	**426**
		HA p28	162–178	162 RNLLWLTGKNGLYPNLS 178	263
		HA p54	316–332	316 IGECPKYVRSAKLRMVT 332	191
		HA p57	334–350	334 LRNIPSIQSRGLFGAIA 350	243
		**HA p66**	**386–402**	**386 NAINGITNKVNSVIEKM 402**	**428**

## Supplementary Materials

The following are available online at https://www.mdpi.com/2076-0817/8/4/220/s1, Table S1: Peptides from H1N1 A/New Caledonia/20/99 and their corresponding amino acid sequence obtained from BEI, Table S2: Each mouse strain including those without epitopes >125 spots/million CD4 T cells. Those peptides in red are those included in Table 1, with the bold corresponding to those with ≥300 spots/million.
