# Supplementary material for: Peptide Epitope Hot Spots of CD4 T Cell Recognition Within Influenza Hemagglutinin During the Primary Response to Infection
Source: Pathogens. 2019 Nov 5;8(4):220. doi: 10.3390/pathogens8040220 (PMC6963931; doi:10.3390/pathogens8040220)
Supplement: Supplementary file 1 [file pathogens-08-00220-s001.zip › pathogens-620523-supplementary/Supplementary Table 2 cones KR 100219.pdf]

Mouse Strain	MHC Haplotype	Peptide	Amino Acids	Sequence	Spots per 10^6 cells
**A/J**	**H-2^a**	**HA p21**	**120-136**	**120 EQLSSVSSFERFEIFPK 136**	**474**
		HA p23	132-148	132 EIFPKESSWPNHTVTGV 148	72
		**HA p30**	**174-190**	**174 YPNLSKSYVNNKEKEVL 190**	**374**
		HA p33	192-208	192 LWGVHHPPNIGNQRALY 208	90
		HA p37	215-231	215 VSVVSSHYSRRFTPEIA 231	236
		HA p43	250-266	250 LEPGDTIIFEANGNLIA 266	74
		HA p48	280-296	280 GIITSNAPMDECDAKCQ 296	81
		HA p52	304-320	304 SSLPFQNVHPVTIGECP 320	116
		HA p56	328-344	328 LRMVTGLRNIPSIQSRG 344	271
		**HA p61**	**358-374**	**358 TGMVDGWYGYHHQNEQG 374**	**507**
		**HA p64**	**375-391**	**375 SGYAADQKSTQNAINGI 391**	**351**
		**HA p68**	**398-414**	**398 VIEKMNTQFTAVGKEFN 414**	**640**
		HA p71	416-432	416 LERRMENLNKKVDDGFL 432	83
		HA p78	458-474	458 VKNLYEKVKSQLKNNAK 474	33
		HA p84	492-508	492 MESVKNGTYDYPKYSEE 508	99
		HA p90	527-543	527 YQILAIYSTVASSLVLL 543	0
**B10.PL**	**I-A^u**	HA p15	84-100	84 CELLISKESWSYIVETP 100	51
		HA p25	144-160	144 TVTGVSASCSHNGKSSF 160	124
		**HA p54**	**316-332**	**316 IGECPKYVRSAKLRMVT 332**	**369**
		HA p55	322-338	322 YVRSAKLRMVTGLRNIP 338	289
		HA p65	380-396	380 DQKSTQNAINGITNKVN 396	62
		HA p79	464-479	464 KVKSQLKNNAKEIGNG 479	105
C57BL/10	H-2^b	HA p16	90-106	90 KESWSYIVETPNPENGT 106	100
		HA p35	203-219	203 NQRALYHTENAYVSVVS 219	119
		HA p52	304-320	304 SSLPFQNVHPVTIGECP 320	93
**HLA-DQ8**	**DQ8**	HA p14	78-94	78 ILGNPECELLISKESWS 94	136
		HA p18	102-118	102 PENGTCYPGYFADYEEL 118	69
		HA p21	120-136	120 EQLSSVSSFERFEIFPK 136	62
		HA p47	274-290	274 SRGFGSGIITSNAPMDE 290	96
		HA p48	280-296	280 GIITSNAPMDECDAKCQ 296	155
		HA p68	398-414	398 VIEKMNTQFTAVGKEFN 414	114
		HA p77	452-468	452 DFHDSNVKNLYEKVKSQ 468	59
**HLA-DR1**	**DR1**	HA p8	43-58	43 VTHSVNLLEDSHNGKL 58	45
		HA p22	126-142	126 SSFERFEIFPKESSWPN 142	101
		HA p23	132-148	132 EIFPKESSWPNHTVTGV 148	62
		HA p27	156-172	156 GKSSFYRNLLWLTGKNG 172	167
		HA p28	162-178	162 RNLLWLTGKNGLYPNLS 178	163
		HA p33	192-208	192 LWGVHHPPNIGNQRALY 208	39

		HA p35	203-219	203 NQRALYHTENAYVSVVS 219	159
		HA p43	250-266	250 LEPGDTIIFEANGNLIA 266	111
		HA p44	256-272	256 IIFEANGNLIAPWYAFA 272	46
		HA p45	262-278	262 GNLIAPWYAFALSRGFG 278	86
		HA p64	375-391	375 SGYAADQKSTQNAINGI 391	118
		HA p67	392-408	392 TNKVNSVIEKMNTQFTA 408	90
		HA p68	398-414	398 VIEKMNTQFTAVGKEFN 414	75
		HA p74	434-450	434 IWTYNAELLVLLENERT 450	128
		HA p75	440-456	440 ELLVLLENERTLDFHDS 456	146
		HA p89	522-538	522 ESMGVYQILAIYSTVAS 538	44
HLA-DR4	DR4	HA p35	203-219	203 NQRALYHTENAYVSVVS 219	51
		HA p56	328-344	328 LRMVTGLRNIPSIQSRG 344	52
		HA p65	380-396	380 DQKSTQNAINGITNKVN 396	39
		HA p79	464-479	464 KVKSQLKNNAKEIGNG 479	36
		HA p94	550-565	550 SFWMCSNGSLQCRICI 565	35
**BALB/c**	**H-2^{d}**	HA p12	66-82	66 PLQLGNCSVAGWILGNP 82	105
		HA p21	120-136	120 EQLSSVSSFERFEIFPK 136	146
		HA p22	126-142	126 SSFERFEIFPKESSWPN 142	151
		HA p37	215-231	215 VSVVSSHYSRRFTPEIA 231	130
		HA p56	328-344	328 LRMVTGLRNIPSIQSRG 344	112
		HA p66	386-402	386 NAINGITNKVNSVIEKM 402	124
**SJL**	**H-2^{s}**	**HA p21**	**120-136**	**120 EQLSSVSSFERFEIFPK 136**	**306**
		**HA p22**	**126-142**	**126 SSFERFEIFPKESSWPN 142**	**663**
		**HA p23**	**132-148**	**132 EIFPKESSWPNHTVTGV 148**	**300**
		HA p24	138-154	138 SSWPNHTVTGVSASCSH 154	91
		**HA p25**	**144-160**	**144 TVTGVSASCSHNGKSSF 160**	**426**
		HA p28	162-178	162 RNLLWLTGKNGLYPNLS 178	263
		HA p38	221-237	221 HYSRRFTPEIAKRPKVR 237	84
		HA p53	310-326	310 NVHPVTIGECPKYVRSA 326	123
		HA p54	316-332	316 IGECPKYVRSAKLRMVT 332	191
		HA p55	322-338	322 YVRSAKLRMVTGLRNIP 338	93
		HA p57	334-350	334 LRNIPSIQSRGLFGAIA 350	243
		HA p65	380-396	380 DQKSTQNAINGITNKVN 396	61
		**HA p66**	**386-402**	**386 NAINGITNKVNSVIEKM 402**	**428**

Supplemental Table 2: Each mouse strain including those without epitopes >125 spots/million CD4 T cells. Those peptides in red are those included in Table 1, with the bold corresponding to those with ≥300 spots/million.
