# Supplementary material for: Peptide Epitope Hot Spots of CD4 T Cell Recognition Within Influenza Hemagglutinin During the Primary Response to Infection
Source: Pathogens. 2019 Nov 5;8(4):220. doi: 10.3390/pathogens8040220 (PMC6963931; doi:10.3390/pathogens8040220)
Supplement: Supplementary file 1 [file pathogens-08-00220-s001.zip › pathogens-620523-supplementary/SupplementaryTable 1 cones KR 100219.pdf]

Peptide Number	Amino Acids	Peptide Sequence
p1	1-17	1-MKAKLLVLLCTFTATYA-17
p2	7-23	7-VLLCTFTATYADTICIG-23
p3	13-29	13-TATYADTICIGYHANNS-29
p4	19-35	19-TICIGYHANNSTDTVDT-35
p5	25-41	25-HANNSTDTVDTVLEKNV-41
p6	31-47	31-DTVDTVLEKNVTVTHSV-47
p7	37-53	37-LEKNVTVTHSVNLLEDS-53
p8	43-58	43-VTHSVNLLEDSHNGKL-58
p9	48-64	48-NLLEDSHNGKLCLLKGI-64
p10	54-70	54-HNGKLCLLKGIAPLQLG-70
p11	60-76	60-LLKGIAPLQLGNCSVAG-76
p12	66-82	66-PLQLGNCSVAGWILGNP-82
p13	72-88	72-CSVAGWILGNPECELLI-88
p14	78-94	78-ILGNPECELLISKESWS-94
p15	84-100	84-CELLISKESWSYIVETP-100
p16	90-106	90-KESWSYIVETPNPENGT-106
p17	96-112	96-IVETPNPENGTCYPGYF-112
p18	102-118	102-PENGTCYPGYFADYEEL-118
p19	108-124	108-YPGYFADYEELREQLSS-124
p20	114-130	114-DYEELREGLSSVSSFER-130
p21	120-136	120-EQLSSVSSFERFEIFPK-136
p22	126-142	126-SSFERFEIFPKESSWPN-142
p23	132-148	132-EIFPKESSWPNHTVTGV-148
p24	138-154	138-SSWPNHTVTGVSASCSH-154
p25	144-160	144-TVTGVSASCSHNGKSSF-160
p26	150-166	150-ASCSHNGKSSFYRNLLW-166
p27	156-172	156-GKSSFYRNLLWLTGKNG-172
p28	162-178	162-RNLLWLTGKNGLYPNLS-178
p29	168-184	168-TGKNGLYPNLSKSYVNN-184
p30	174-190	174-YPNLSKSYVNNKEKEVL-190
p31	180-196	180-SYVNNKEKEVLVLWGVH-196
p32	186-202	186-EKEVLVLWGVHHPPNIG-202
p33	192-208	192-LWGVHHPPNIGNQRALY-208
p34	198-214	198-PPNIGNQRALYHTENAY-214
p35	203-219	203-NQRALYHTENAYVSVVS-219
p36	209-225	209-HTENAYVSVVSSHYSRR-225
p37	215-231	215-VSVVSSHYSRRFTPEIA-231
p38	221-237	221-HYSSRRFTPEIAKRPKVR-237
p39	227-243	227-TPEIAKRPKVRDQEGRI-243
p40	233-249	233-RPKVRDQEGRINYYWTL-249
p41	238-254	238-DQEGRINYYWTLLEPGD-254
p42	244-260	244-NYYWTLLEPGDTIIFEA-260
p43	250-266	250-LEPGDTIIFEANGNLIA-266
p44	256-272	256-IIFEANGNLIAPWYAFA-272
p45	262-278	262-GNLIAPWYAFALSRGFG-278
p46	268-284	268-WYAFALSRGFGSGIITS-284
p47	274-290	274-SRGFGSGIITSNAPMDE-290
p48	280-296	280-GIITSNAPMDECDAKCQ-296
p49	286-302	286-APMDECDAKCQTPQGAI-302
p50	292-308	292-DAKCQTPQGAINSSLPF-308

p51	298-314	298-PQGAINSSLPFQNVHPV-314
p52	304-320	304-SSLPFQNVHPVTIGECP-320
p53	310-326	310-NVHPVTIGECPKYVRSA-326
p54	316-332	316-IGECPKYVRSAKLRMVT-332
p55	322-338	322-YVRSAKLRMVTGLRNIP-338
p56	328-344	328-LRMVTGLRNIPSIQSRG-344
p57	334-350	334-LRNIPSIQSRGLFGAIA-350
p58	340-356	340-IQSRGLFGAIAGFIEGG-356
p59	346-362	346-FGAIAGFIEGGWTGMVD-362
p60	352-368	352-FIEGGWTGMVDGWYGYH-368
p61	358-374	358-TGMVDGWYGYHHQNEQG-374
p62	364-380	364-WYGYHHQNEQGSGYAAD-380
p63	369-385	369-HQNEQGSGYAADQKSTQ-385
p64	375-391	375-SGYAADQKSTQNAINGI-391
p65	380-396	380-DQKSTQNAINGITNKVN-396
p66	386-402	386-NAINGITNKVNSVIEKM-402
p67	392-408	392-TNKVNSVIEKMNTQFTA-408
p68	398-414	398-VIEKMNTQFTAVGKEFN-414
p69	404-420	404-TQFTAVGKEFNKLERRM-420
p70	410-426	410-GKEFNKLERRMENLNKK-426
p71	416-432	416-LERRMENLNKKVDDGFL-432
p72	422-438	422-NLNKKVDDGFLDIWTYN-438
p73	428-444	428-DDGFLDIWTYNAELLVL-444
p74	434-450	434-IWTYNAELLVLLENERT-450
p75	440-456	440-ELLVLLENERTLDFHDS-456
p76	446-462	446-ENERTLDFHDSNVKNLY-462
p77	452-468	452-DFHDSNVKNLYEKVKSQ-468
p78	458-474	458-VKNLYEKVKSQLKNNAK-474
p79	464-479	464-KVKSQLKNNAKEIGNG-479
p80	469-485	469-LKNNAKEIGNGCFEFYH-485
p81	475-490	475-EIGNGCFEFYHKCNNE-490
p82	480-496	480-CFEFYHKCNNECMESVK-496
p83	486-502	486-KCNNECMESVKNGTYDY-502
p84	492-508	492-MESVKNGTYDYPKYSEE-508
p85	498-514	498-GTYDYPKYSEESKLNRE-514
p86	504-520	504-KYSEESKLNREKIDGVK-520
p87	510-526	510-KLNREKIDGVKLESMGV-526
p88	516-532	516-IDGVKLESMGVYGILAI-532
p89	522-538	522-ESMGVYQILAIYSTVAS-538
p90	527-543	527-YQILAIYSTVASSLVLL-543
p91	533-549	533-YSTVASSLVLLVSLGAI-549
p92	539-555	539-SLVLLVSLGAISFWMCS-555
p93	545-560	545-SLGAISFWMCSNGSLQ-560
p94	550-565	550-SFWMCSNGSLQCRICI-565

Supplemental Table 1: Peptides from H1N1 A/New Caledonia/20/99 and their corresponding amino acid sequence obtained from BEI.
